# Resource Requirements of the Pacific Leatherback Turtle Population

**DOI:** 10.1371/journal.pone.0045447

**Published:** 2012-10-05

**Authors:** T. Todd Jones, Brian L. Bostrom, Mervin D. Hastings, Kyle S. Van Houtan, Daniel Pauly, David R. Jones

**Affiliations:** 1 Department of Zoology, University of British Columbia, Vancouver, British Columbia, Canada; 2 NOAA Fisheries, Pacific Islands Fisheries Science Center, Honolulu, Hawaii, United States of America; 3 Conservation and Fisheries Department, Ministry of Natural Resources and Labour, Government of the British Virgin Islands, Road Town, Tortola, British Virgin Islands; 4 Nicholas School of the Environment and Earth Sciences, Duke University, Durham, North Carolina, United States of America; 5 Sea Around Us Project, Fisheries Centre, University of British Columbia, Vancouver, British Columbia, Canada; Monash University, Australia

## Abstract

The Pacific population of leatherback sea turtles (*Dermochelys coriacea*) has drastically declined in the last 25 years. This decline has been linked to incidental capture by fisheries, egg and meat harvesting, and recently, to climate variability and resource limitation. Here we couple growth rates with feeding experiments and food intake functions to estimate daily energy requirements of leatherbacks throughout their development. We then estimate mortality rates from available data, enabling us to raise food intake (energy requirements) of the individual to the population level. We place energy requirements in context of available resources (i.e., gelatinous zooplankton abundance). Estimated consumption rates suggest that a single leatherback will eat upward of 1000 metric tonnes (t) of jellyfish in its lifetime (range 924–1112) with the Pacific population consuming 2.1×10^6^ t of jellyfish annually (range 1.0–3.7×10^6^) equivalent to 4.2×10^8^ megajoules (MJ) (range 2.0–7.4×10^8^). Model estimates suggest 2–7 yr-old juveniles comprise the majority of the Pacific leatherback population biomass and account for most of the jellyfish consumption (1.1×10^6^ t of jellyfish or 2.2×10^8^ MJ per year). Leatherbacks are large gelatinous zooplanktivores with consumption to biomass ratios of 96 (up to 192 if feeding strictly on low energy density Cnidarians); they, therefore, have a large capacity to impact gelatinous zooplankton landscapes. Understanding the leatherback's needs for gelatinous zooplankton, versus the availability of these resources, can help us better assess population trends and the influence of climate induced resource limitations to reproductive output.

## Introduction

The nesting population of the endangered leatherback sea turtle (*Dermochelys coriacea*) (United States Endangered Species Act of 1973) in the eastern Pacific Ocean is perhaps the most imperiled of any marine turtle population. The documented declines in population numbers [1], [2] are thought to be a result of direct exploitation of adults and egg harvesting [3], to incidental capture in commercial and artisanal fisheries [4], [5], and to climate-induced fluctuations or ocean basin differences in resource availability [6], [7]. Despite extensive research, there still remains a lack of data on population size, distribution, and resource requirements of leatherbacks that are required to manage this endangered species [8] beyond the nesting beaches. Managing fisheries interactions and understanding climate change impacts, however, require knowledge of marine turtle resource needs. Daily intake needs of the individual versus resource accessibility influence movement and distribution patterns. Understanding the effects of climate (e.g., climatic change, El Niño Southern Oscillation (ENSO)) on resource availability requires knowledge of baseline resource requirement of the individual throughout ontogeny and at the population level.

Food requirement is perhaps the most useful measure for understanding constraints on bioenergetics because it represents the energy that has to be derived from resources available in the animal's habitat [9]. Extrapolating individual, daily energy demands to an entire population allows an understanding of the dynamics involved in determining animal abundance and distribution [10]. For instance, Wallace *et al*. [6] calculated the costs associated with nesting by North Atlantic and eastern Pacific leatherbacks and suggested that limited resource availability constrained energy allocation to reproduction in eastern Pacific leatherbacks, therefore lowering their reproductive output.

Leatherbacks are obligate jelly (gelatinous zooplankton) consumers throughout their ontogeny [11], [12]. Witt *et al*. [13] used continuous plankton recorder survey data to map gelatinous zooplankton landscapes in the North Atlantic in conjunction with sea surface temperature (SST) to infer potential hotspots for leatherback foraging. Shillinger *et al.* [14] studied the oceanographic information surrounding directed leatherback movements in the South Pacific Gyre to better understand preferred habitat (assumed as areas of dense gelatinous zooplankton). Further studies have determined how climatic patterns (e.g., ENSO) affect the yearly abundance of resources in the eastern Pacific [7], [15], [16], thus causing variable recruitment rates among the leatherback population. Satellite tracking suggests that leatherbacks follow jellyfish distributions during their post-nesting migrations [17], [18], [19]. However, basic data regarding daily energetic demands or food (jellyfish) intake rates are lacking and generally limited to inferred metabolic rates from oxygen consumption data on turtle hatchlings [20], [21] or nesting females on beaches [22], [23], with one study using doubly labeled water to estimate field metabolic rate of inter-nesting females [24] (see Wallace and Jones [25] for review). To our knowledge, only two reports have documented the food intake rate (jellyfish consumption) of adult leatherbacks in the wild [26], [27], based on the observations of the leatherbacks foraging at or near the surface (off the French Coast and Solomon Islands, respectively) and a single report observing post-hatchlings foraging on gelatinous diet items within 20 meters of the surface [12]. Therefore, it seems appropriate to conclude that existing data leave large gaps in our knowledge of the ontogeny of energy requirements across all life-history stages of leatherbacks.

This study determined: 1) food intake (daily energy requirements) for individual leatherbacks from growth and food conversion rates [28] of a captive stock [29]; 2) leatherback population biomass and population food consumption rates (Pacific population) by combining measured growth and food intake rates with estimates of mortality [30]; 3) high and low estimates of food intake, population biomass, and population food intake rates by Monte Carlo simulations; and 4) validity of the output of our model with metabolic data from the literature.

## Methods

### Energetics Study

Twenty hatchlings (emergence July 2, 2005) were transported from Tortola, British Virgin Islands (BVI) to the Animal Care Center, Department of Zoology, University of British Columbia (Canada permit# CA05CWIM0039, BVI certificate# CFD062005). Turtles were maintained in large oval tanks (5 m long×1.5 m wide×0.3 m deep) containing ∼2500 l of recirculated/filtered salt water. As the turtles grew in size, header tanks were added that doubled or tripled the active volume of filtered water per turtle. The water temperature was maintained at 24±1°C. Four fluorescent fixtures (40 W UVA/B; Exo Terra Repti-Glo® 8, Mansfield Massachusetts) suspended 0.5 m above each tank provided full spectrum radiation for 12 hours per day; each tank was also exposed to ambient light. Water quality was maintained between the following levels: pH = 8.0–8.3; salinity = 28–33 ppt; and ammonia < 0.1 mg^−1^. All turtles were housed and maintained for research purposes and all animal care standards of the Canadian Council for Animal Care (CCAC) and the UBC Animal Care Committee were met (UBC Animal Care Protocol: A04-0323). The complete husbandry protocols used in this study are provided in Jones *et al*. [29].

The diet of wild leatherbacks consists solely of gelatinous zooplankton (e.g., jellyfish, ctenophores). Throughout the study period we made a diet, which could be made readily and consistently with respect to energy and water content, that replicated their natural diet in terms of texture and that the turtles would accept. The diet was made up of squid (Pacific Ocean squid, *Loligo* sp.; mantle, arms, and tentacles only), vitamins (Zoo Med Reptavite™, San Luis Obispo, California), and calcium (Zoo Med Rep-Cal™), blended with unflavored gelatin in hot water. The mixture was poured into shallow trays and refrigerated. The solidified diet was cut into strips for ease of feeding and weighing. Turtles were fed 3 to 5 times daily to satiation during the first 2 months of age, and 3 times daily to satiation when >2 months of age. The food for each leatherback was weighed prior to (and the residue after) feeding to obtain food intake (Ek-1200 A; Stites Scale Inc., 3424 Beekman Street, Cincinnati, OH45223).

Food samples were taken at random from a mixture of several food batches and dried in a desiccating oven at 60°C for 48 to 72 hours to determine dry-to-wet-weight ratios. Dried homogenized samples were analyzed for energy content by bomb calorimetry (Parr Instrument Co., 211 Fifty Third Street, Moline, Illinois 61265). The food had a water content of 90% and an energy content of 20.16 kJ g^−1^ (SD 0.58) dry mass (DM). The former almost matched the water content of jellyfish, which can be upwards of 96% [31], while the latter was 4 to 10 times greater than the energy content of common gelatinous prey items of leatherbacks (range 2.0 to 5.0 kJ g^−1^ DM: [31], [32], [33], [34]). Food intake values were converted to energy intake using the gross energy content of the food. Based on energy results, the equivalent total mass of jellyfish that would have been consumed by leatherbacks was derived by multiplying the mass of the consumed gelatin diet by 10 (e.g., using an average energy content of 4 kJ g^−1^ DM and 95% water content from the jellyfish studies above would equate to 0.2 kJ g^−1^ wet mass (WM), whereas the gelatin diet has an energy content of 2.0 kJ g^−1^ WM; a tenfold difference).

An Ek-1200 A scale was used weekly to weigh turtles that ranged from hatchling to a body mass of 1.2 kg (±0.001 kg), and an ADAM CPW-60 scale (Dynamic Scales, 1466 South 8th Street, Terre Haute, IN 47802) was used to weigh turtles with body masses >1.2 kg (±0.02 kg).

### Data Analysis and Modeling

The von Bertalanffy growth function (VBGF; see Ricker [30]) was fitted to our growth data
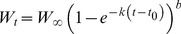
(1)where *W_t_* is the predicted mass (kg) at age *t*, *W_∞_* is the mean mass the adults in the population asymptotically approach, *k* is a growth parameter (not a growth rate) of dimension time^−1^, *t_0_* is the theoretical age at mass = 0, and *b* is the exponent from a length-mass relationship of the form:
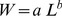
(2)where *W* is mass in kg, *L* is length in cm straight carapace length (SCL), *a* is a multiplicative parameter and *b* an exponent usually having a value close to 3. Coefficients *a* and *b* were estimated as 2.14×10^−4^ (SEM 1.4×10^−5^) and 2.86 (SEM 0.01), respectively from a length-mass relationship [29]. The first derivative of the VBGF (*dW_t_*/*dt*) of the form:
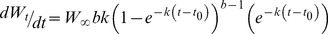
(3)represents the growth rate and declines linearly with mass, reaching zero at *W_∞_*.

Feeding experiments allow calculation of gross food conversion efficiency *K_1_* [28]. It can be calculated by dividing body mass increase over a specified time by the rate of food consumption (*F_1_*), or:
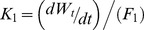
(4)


Weekly measurements of mass gain and food consumed by individual leatherbacks were used to determine *K_1_*. Estimates of *K_1_*were assigned as the average of the animal's mass over the time increment, (*W_i_*+*W_j_*/2), and these values were related to the mass of the animals by the following function [28]:
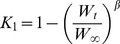
(5)where *β* is a constant. It is a property of the model that *K_1_* approaches 0 as *W_t_* approaches *W_∞_*. Data in eq. 5 were fit by linear regression after log-log transformation:
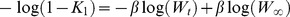
(6)


The rate of food consumption as a function of age (*F*_1,*t*_) can be determined by rearranging eq. 4:
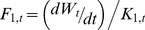
(7)where *K*_1,*t*_ is the animal's conversion efficiency as a function of age (determined by combining equations 1 & 5). Substituting these equations into eq. 7, food consumption (*F*_1,*t*_) can be plotted as:
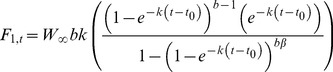
(8)providing the food intake of an animal at any age.

Energy is consumed by an animal in the form of food and that food energy is either stored or used by the animal in external work or internal heat production [9], [35], [36]. Growing animals can be considered to be in a positive energy balance, with the amount of energy taken in being greater than expended. The extra energy is primarily stored as adipose tissue or glycogen, or used in somatic growth. Mature animals are probably in a neutral energy balance where food intake more closely matches the amount of energy expended [36]. The fate of ingested food energy (*C*) can be expressed by the following equation [9], [35]:
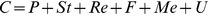
(9)where *P* = production (i.e., growth), *St* = storage (e.g., glycogen stores in cells), *Re* = respiration (i.e., metabolic rate), *F* = feces, *Me* = methane gas produced in the alimentary tract and *U* = excretion (i.e., nitrogenous waste). The terms *P*, *St*, *Re*, and *U* refer to the apparent absorption (*A*); apparent because secretions are added to the gut, thus *A* is the net absorbed energy. The efficiency of this process is known as the assimilation efficiency (*AE*) and depicted as a percentage (%).

By combining food consumption (*F*_1,*t*_) , conversion rate (1–*K*_1_), and assimilation efficiency (*AE*) we can determine metabolic rate (*MR*) as follows:
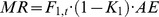
(10)


Estimates of the biomass of the population of leatherbacks and their food consumption can be determined with known mortality estimates (throughout life cycle). To this end, we used published data on leatherback nesting ecology in the Pacific Ocean (Atlantic and Indian Ocean data are incomplete) to determine the number of nesting females per year [2], [37], [38], [39] and then multiplied this by the following: nests per female [40], eggs per nest [40], [41], % hatching success [37], [41], [42], emergence rate [43], first day survivorship on crawl to water [44], and first day survivorship during frenzy period swim [45]. This resulted in an estimate of the number of hatchlings that enter the Pacific on average each year (Table 1). Survivorship during the first year was assumed as 25% of the total number of hatchlings from day 2 through day 365 [37]. High and low number of new recruits (first time nesters) entering the adult population each year was estimated by (i) taking % first-time nesters each year (0.50±0.016) estimated using a binomial proportion from the nesting data presented in Santadrian-Tomillo et al. [39] and multiplying by total number of nesting females (Table 1) per year [2], [37], [38], [39]; (ii) multiplying this by 1.25 for the eastern Pacific population and 2 for the western Pacific population, i.e., assuming a 4∶1 and 1∶1 female to male ratio, respectively . Hatchling sex ratios ranged from 64% to 100% female from the 1993 to 2007 nesting seasons at Playa Grande Beach, Costa Rica [46], [47]. Less data is available for the western Pacific, while there is evidence for female bias [48] the region may also have less of a female skew due to heavy rainfall as found in the Atlantic [49]. If adult sex ratios are more or less female skewed than we have modeled here this would lower or raise our estimate of the adult population but would not change the calculated number of hatchings entering the ocean each year. We used 2 standard errors of the mean (SEM) from the averages given (or range if error not given) for nests per year, eggs per nest, etc… to obtain best and worst case scenarios for mortality estimates. These data were then matched to a mortality equation [30] of the form: 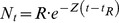
(11)where *N_t_* is the number of individuals living at age *t*, *R* is the number of recruits, *Z* is the instantaneous mortality rate where (ln 2/*Z*) gives the half-life (i.e. time when there will be half the recruited number of turtles), and *t_R_* refers to the age at recruitment or in our case hatching (*t_R_* = 0), yearlings (*t_R_* = 1), and age-at-maturity (*t_R_* = 16) [29]. Annual mortality rate (*A*) can be determined from eq. 11 by allowing *M* = 1–*e^−Z^*, and annual survivor rate (*S*) can be determined by allowing, *S* = *e ^−Z^*.

**Table 1:** Total number of hatchlings entering the Pacific Ocean each year calculated from nesting ecology data from the literature.

Variable	low	mean	high	Reference
nesting females per year eastern Pacific (EP)	248	248	248	[2], [39]
nests per year	4.3	6.1	7.9	[40]
number eggs per nest	61.3	64.1	66.9	[40]
hatching success	0.39	0.47	0.55	[37], [41], [42]
emergence rate	0.51	0.76	1	[43]
first day survivorship: beach	0.78	0.83	0.87	[44]
first day survivorship: water	0.64	0.69	0.74	[45]
Total hatchlings (EP):	**6,491**	**19,837**	**46,411**	
nesting females per year				
western Pacific (WP)	1113	1113	1113	[38]
nests per year	4.3	6.1	7.9	[40]
number eggs per nest	73.2	77.9	82.6	[41]
hatching success	0.39	0.47	0.55	[37], [41], [42]
emergence rate	0.51	0.76	1	[43]
first day survivorship: beach	0.78	0.83	0.87	[44]
first day survivorship: water	0.64	0.69	0.74	[45]
total hatchlings (WP):	**34,784**	**108,193**	**257,167**	
total hatchling production for the Pacific:	**41,275**	**128,031**	**303,578**	

Low and high values are ±2 standard errors of the mean (SEM), or from a range when SEM not given.

Multiplying food consumption as a function of age (eq. 8) by number of turtles alive at age (eq. 11) provides age-specific food intake per year:
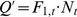
(12)where 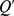 represents the intake of jellyfish in metric tonnes (*t*) per year. The biomass of a turtle cohort at age t (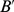), in metric tonnes, can be determined by multiplying turtle body mass (eq. 1) by number of turtles alive at age (eq. 11):
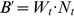
(13)Finally, by dividing the integral of 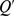 (eq. 12) by the integral of 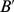 (eq. 13) we obtain the overall quantity of jellyfish consumed per unit biomass of leatherback per year; or how many times the population will consume its own mass in jellyfish:
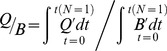
(14)where *Q*/*B* has the units year^−1^.

We used generalized linear models (GLM) to estimate the parameters of eqs. 1, 2, and 6. The parameter estimates were used in turn to model annual dietary consumption across age by using eq. 8. The empirical distributions of parameter estimates from GLM fits, determined from the estimated standard errors, were used in a Monte Carlo (MC) resampling method. Using random samples from distributions of *β*, *b*, and *k*, we computed 10,000 runs of eqs. 8, 12, and 13. The model mean from the average of the MC runs at each time step were determined, which corresponds to the value obtained by simply inputting the GLM reported parameters into eqs. 8, 12, and 13. MC results at each time step were ranked and a 95% confidence interval for each parameter was obtained by excluding the highest and lowest 2.5% of the results. The mean and 95% CI obtained from the MC exercise with eq. 8 were used to model eq. 10.

## Results

### Growth and food consumption

**Figure 1:**
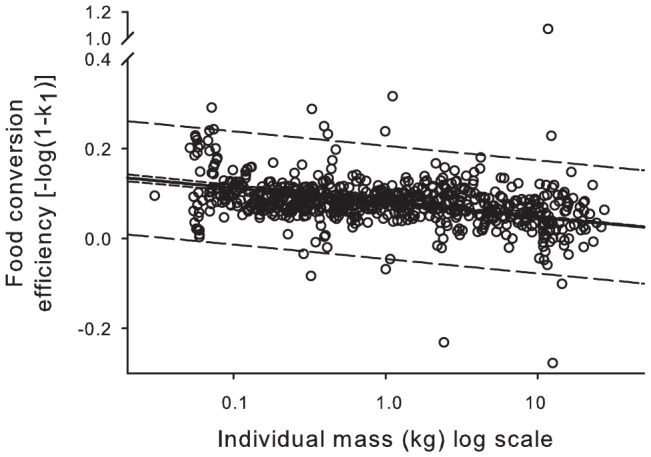
Log-log transformation of food conversion efficiency ‘K_1_’ versus an individual's mass (kg) showing the best-fit curve from GLM with 95% confidence bands (short-dashed lines) and 95% prediction bands (long-dashed lines).

**Figure 2:**
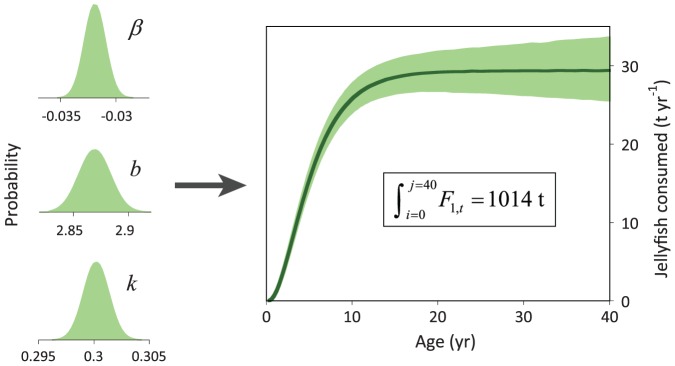
Annual leatherback dietary consumption rates. Three left panels show the empirically estimated probability density functions for the variables *β* (food conversion constant), *b* (scaling exponent), and *k* (growth parameter) which populate the consumption model (right). The solid dark green line on the right plot is the model average, the green shaded area is the 95% CI, obtained from Monte Carlo simulations of the input variables. Integration of the model average suggests that a leatherback will consume 1014 t of jellyfish (range 924–1112) from hatching through adulthood (age of 40 yrs).

Combining equations 1 and 2, using the variation in parameters *a* and *b* as found in Jones et al. [29] to estimate a range in *W_∞_* (267–379), provided a VBGF (in kg) for mass where *k* = 0.299 (SEM 0.001) (*t* = 265.17, *p*<0.0001) and *b* = 2.86 (SEM 0.014) (*t* = 206.03, *p*<0.0001). Food conversion efficiency (*K*_1_) in the form of eq.6 is depicted in Fig. 1, where the slope of the line (*β*) is 0.0328 (SEM 0.001) (*t* = 35.26, *p*<0.0001). Figure 2 plots food consumption as a function of age, incorporating the uncertainty in the estimates of the growth parameters *k* and *b* and food conversion parameter *β*. Integration (computing the area under the black line in Fig. 2) indicates that from hatching, a leatherback will require >310 t of jellyfish (range 291–332) to attain a size characteristic of sexual maturity, assumed as 16 years [29], and will consume 1014 t (range 924–1112) in its lifetime (>3000 times its adult body mass), assuming a longevity of 40 years.

### Metabolic rate and validation of the model

**Figure 3:**
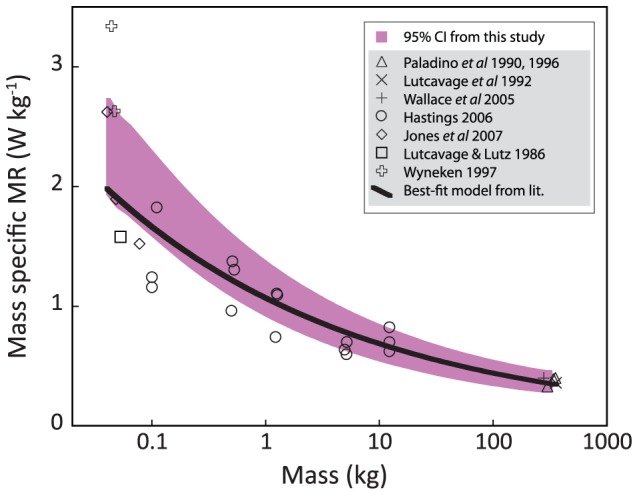
Metabolic rate (MR) (W kg^−1^) determined from food consumption this study (pink shaded area) plotted with metabolic rates of leatherback hatchlings, juveniles, and adults from the literature (symbols). Pink shaded area is the 95% CI estimated from the variability in parameters *β* (food conversion constant), *b* (scaling exponent), and *k* (growth parameter), see Figures 1 and 2. Black solid line is the best-fit model through the literature MR values (given by symbols).

Metabolic rate (*MR*, eq. 10) was estimated by assuming a digestive efficiency of 80% for jellyfish [6], [50] and squid [51], [52], [53] and an additional 80% for assimilation efficiency (total available energy 63%) based on diets of similar protein content [54]. The *MR* results from this study were compared (Fig. 3) with resting and field metabolic rates from the literature [20], [21], [22], [23], [24], [32], [55], [56]. The *MR* determined from food consumption coincides with *MR* determinations from the literature (Fig. 3) giving independent validation of estimates of food intake rates, growth, and food conversion rates.

Further validation is provided from the total energy stored in a leatherback. Using total body water values of 71.2% (includes carapace) for adult leatherbacks (adapted from [24]) and 21.1 kJ g^−1^ DM of homogenized body tissue including carapace [21], an adult leatherback (319 kg; adapted from [29]) is made up of 1938 MJ of energy. Given the average gross food conversion rate of a leatherback from hatching to maturity (*β* from eq. 6, Fig. 1) and the average energy of gelatinous zooplankton, this suggests that a leatherback needs to consume ∼295 t of jellyfish from hatching until reaching age at maturity (16 years, [29]). This estimate falls within the range of our previous calculations (291–332 t of jellyfish, eq. 8, Fig. 2) further lending support to our model of resource requirements in leatherbacks.

### Mortality, food consumption, and biomass of the Pacific population

Combining data on nesting ecology suggests that 128,031 (range 41,27–303,578) hatchlings enter the Pacific Ocean annually from nesting beaches in the eastern and western Pacific (Table 1). Of these, we assumed 25% or 32,338 (range 10,425–76,679) turtles survive their first year, and 1268 (range 1217–1318) of the yearlings survive their juvenile and subadult years and recruit to the adult reproductive population at age 16 yr. Inserting these values into eq. 11 we obtain an abundance estimate of *N_t_* = 128031*e* ^−1.38(*t-0.0027)*^ for the hatchling to yearling stage, *N_t_* = 32338*e* ^-0.216(*t*-1)^ for the yearling to adult stage, and *N_t_* = 1268*e* ^-0.229(*t*-16)^ for adults. The mortality coefficient for the subadult stage was estimated as −ln(1268/32338)/15 = 0.216 yr^−1^ and for adults it was estimated as −ln(1/1268)/31 = 0.229 yr^−1^.

Taking into consideration high and low estimates of annual hatchling production, we obtained the following ranges of estimates for cohort abundance (and corresponding mortality coefficients) during the first year post-hatchling and the period between yearling and sexual maturation (Table 2). For turtles aged 0.0027<*t*<1: *N_t_* = 303578*e* ^−1.38*t*^ and *N_t_* = 41275*e* ^−1.38*t*^; and for turtles aged 1<*t*<16: *N_t_* = 76679*e* ^−0.296(*t*-1)^ and *N_t_* = 10425 *e* ^−0.143(*t*-1)^. These mortality/survival curves correspond to an entire population of 294,088 leatherbacks (range 114,663–628,875) of which 6199 are adults (range 4292–8103). In a given year 0.46% of the population or 1 in 217 turtles (females) nest. For hatchlings, juveniles, and adults, half-lives are 0.5, 3.2, and 3.0 years and annual mortality rates (*A*) are 0.75, 0.19, and 0.20, respectively. These results correspond to annual survival rates of 0.25 for first-year leatherbacks, 0.81 (range 0.74–0.87) for juveniles, and 0.80 (range 0.72–0.84) for adults.

**Table 2:** Computation of turtle abundance, survival, and related parameters based on estimates of hatchling production, new recruits, and sex ratios from the literature.

Variable		Low	Mean	High	References
	Number of hatchlings	41,275	128,031	303,578	
Age: 2.7×10^−3^<t<1	Abundance (t)	*N_t_* = 41275*e* ^−1.38(*t-0.0027)*^	*N_t_* = 128031*e* ^−1.38(*t-0.0027)*^	*N_t_* = 303578*e* ^−1.38(*t-0.0027)*^	
	Annual survival	0.25	0.25	0.25	[37]
	Mortality coefficient yr^−1^	1.38	1.38	1.38	
	Number of yearlings	10,425	32,338	76,679	
Age: 1<t<16	Abundance (t)	*N_t_* = 10425*e* ^−0.143(*t*-1)^	*N_t_* = 32338*e* ^−0.216(*t*-1)^	*N_t_* = 76679*e* ^−0.296(*t*-1)^	
	Annual survival	0.87	0.81	0.76	
	Mortality coefficient yr^−1^	0.143	0.216	0.271	
	Number of recruits to adult pop.	1217	1268	1318	[2], [37], [38], [39]
Age: 16<t	Abundance (t)	*N_t_* = 1217*e* ^−0.333(*t*-16)^	*N_t_* = 1268*e* ^−0.229(*t*-16)^	*N_t_* = 1318*e* ^−0.177(*t*-16)^	
	Annual survival	0.72	0.79	0.84	
	Mortality coefficient yr^−1^	0.333	0.229	0.177	
	Total number of adults in pop.	4292	6199	8103	

**Figure 4:**
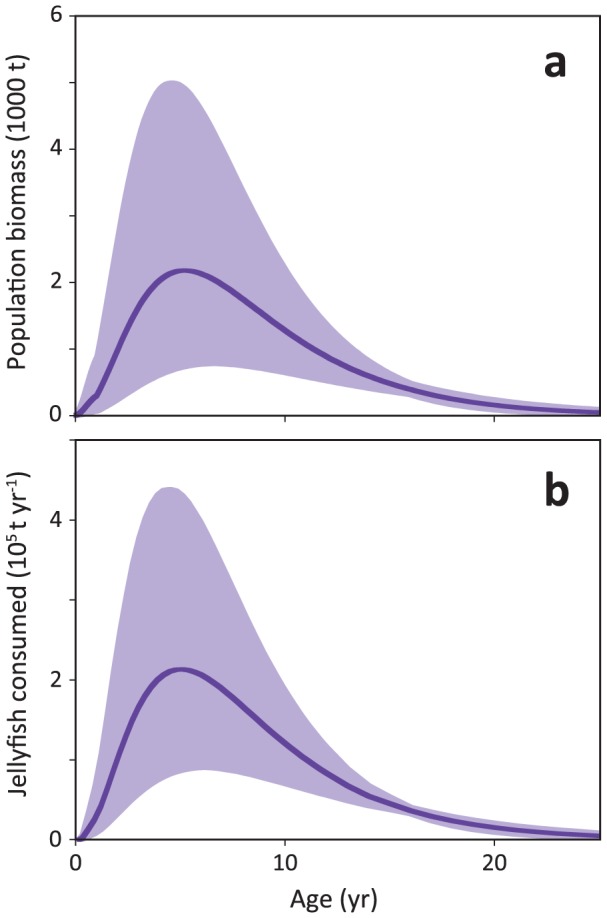
Total Pacific leatherback population biomass (a) determined by multiplying mortality with the VBGF for mass; and consumption rates in metric tonnes of jellyfish per year for the entire Pacific leatherback population (b) determined by multiplying mortality with food consumption rate. Solid dark purple line on plots a and b is the model average, purple shaded area is the 95% CI, obtained from Monte Carlo simulations of the input variables.

The food consumption rate of the Pacific leatherback population (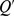, eq. 12) and total leatherback biomass (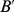, eq. 13) are shown in Fig. 4 as the output of multiplying numerical abundance of leatherbacks (eq. 11) by food consumption rate (eq. 8; Fig. 4b) and by the VBGF for body mass (eq. 1; Fig. 4a), respectively. Integration of these curves shows that the Pacific population consumes 2.1×10^6^ t of jellyfish per year (range 1.0–3.7×10^6^) equivalent to 4.2×10^8^ megajoules (MJ) (range 2.0–7.4×10^8^). Over fifty percent of the jellyfish consumed is being eaten by 2–7-year-old juveniles (1.1×10^6^ t of jellyfish or 2.2×10^8^ MJ per year), whilst adults only account for <9% of the total population consumption (1.8×10^5^ t of jellyfish or 3.6×10^7^ MJ). The 2–7-year-old juveniles also account for most (10,936 t) of the total biomass of the Pacific leatherback population (21,510 t; range 9201–43,198). Immature turtles total 19,955 t, while adults make up less than 10% of the total population biomass (1951 t).

Annual jellyfish consumption per unit biomass, *Q*/*B* (eq. 14), for the Pacific population is estimated to be 96 (range 87–113), thus the population would consume, on average, 96 times its biomass in jellyfish each year. Averaged over the entire population age structure, this is equivalent to leatherbacks eating 26% of their body mass in jellyfish per day; the % consumption per day would be higher in growing juveniles and lower in adults which have growth rates near zero. For the latter, our data suggest that on average adult leatherbacks (250–450 kg) consume 65–117 kg of jellyfish per day to meet daily energetic demands.

## Discussion

Estimated consumption rates suggest that an individual leatherback will eat upward of 1014t of jellyfish (range 924–1112) in its lifetime. To our knowledge, this quantitative analysis is the first study to determine the energy requirements of a marine turtle in such a holistic fashion, by integrating consumption from hatchling stage through to an individual's maturity. Our technique was validated by deriving MR values from food consumption and comparing these to metabolic rate measurements in the literature. The excellent agreement between our estimates and directly recorded values clearly supported our use of the computational method to help elucidate marine turtle energetics.

Bradshaw *et al*. [57] determined field metabolic rate (FMR) by analyzing dive profiles of leatherbacks in conjunction with known oxygen stores [22]. They concluded that leatherbacks dive within but close to their aerobic dive limit, thus dividing total oxygen stores by mean length of the extended dives resulted in FMRs of 21 kJ kg^−1^ day^−1^ [57]. This value falls within the lower range of MR (as to be expected for diving) measured from nesting leatherbacks by respirometry [22], [23], [55], doubly labeled water (DLW) derived FMR [24], and through computational analysis of food conversion (this study). The study by Bradshaw *et al*. [57] and this study highlight the relevance and applicability of computational modeling to deriving estimates of FMR and ultimately resource requirements.

Our estimates of jellyfish consumption for adult leatherbacks (65–117 kg) agreed with findings in a recent study by Wallace *et al.* [6] which showed that eastern Pacific and North Atlantic leatherbacks require 70 to 90 kg of jellyfish per day and up to 87 to 113 kg depending on nesting remigration intervals. Earlier reports had indicated that leatherbacks must consume their body mass of jellyfish each day [32]. However, these estimates were scaled from the energetic demands of hatchlings in which the costs of growth are high. Davenport [58] suggested that leatherbacks consume 50% of their body mass per day in jellyfish, based on direct observations of foraging leatherbacks [26] and taking into account the energetic cost of warming cold gelatinous prey items. We found that leatherbacks must consume 26% of their body mass in jellyfish per day (averaged over their entire population structure) to meet maintenance or routine metabolic rate [59].

How does this level of predation by leatherbacks compare to natural abundance levels of jellyfish? Declines in many fish stocks and the proliferation of jellyfish have been linked to fishing down [60] or through [61] marine food webs. With the removal of their top pelagic predators, studies have postulated that jellyfish outcompete fish for resources [62]. As jellyfish abundance increases, the impacts on fish are compounded because jellyfish prey on fish eggs and larvae [63]. Warming climatic patterns and eutrophication may further be fueling the rapid and vast expansion of jellyfish numbers [64]. Reported global increases in jellyfish, however, may not represent an increase in leatherback prey availability. In a recent study Lynam *et al*. [63] reported high densities of Cnidarians *Chrysaora hysoscella* and *Aequorea forskalea*. *C. hysoscella* (class Scyphozoa) is a known forage item of leatherbacks [65] but *A. forskalea*, a Hydrozoa which made up 99% of the densities reported in Lynam *et al*. [63], is not a major component of the leatherbacks' diet [66]. To date we only know of two reports indicating that leatherbacks forage on Hydrozoa (e.g., Leptomedusae & Siphonophorae), and further it was suggested that the presence of *Aequorea spp*. (order Leptomedusae) in the leatherback alimentary tract may be a result of contamination as Scyphozoa (known leatherback prey items) feed on *Aequorea spp.* [66], [67]. Leatherbacks have also been reported to feed on pyrosomes [33], ctenophores and gelatinous fish egg sacs [12]. Squid, octopus, and fish have been noted in the alimentary tract of three leatherbacks caught in fishing gear [68], [69], [70]. The majority of reported leatherback prey items, however, consist of the phylum Cnidaria, class Scyphozoa (i.e., true jellyfish) including *Aurelia spp*., *Catostylus spp*., *Chrysaora spp*., *Cyanea spp*., *Linuche spp*., *Pelagia spp*., *Rhizostoma spp*., and *Stomolophus spp*. [12], [26], [27], [58], [65], [66], [71], [72], [73]. While the gelatinous diet of leatherbacks seems varied across several phyla and classes, it is unknown if current increases in jellyfish abundance are of consequence to leatherback populations as many of the jellyfish blooms are invasive species [64], not known to currently be eaten by leatherbacks, and mostly coastal in nature [13], [74].

Lynam *et al*. [63] have shown that coastal densities of jellyfish in the Atlantic Ocean off Africa are up to 105 t km^−2^. If similar densities of known leatherback prey were to occur in the Pacific Ocean, the entire leatherback population's yearly consumption could be obtained from 20,163 km^2^. As previously noted, however, only 1% of the gelatinous zooplankton densities reported by Lynam *et al*. [63] were of known leatherback prey. Lilley *et al*. [75] estimated global jellyfish biomass by converting survey data (e.g., tow data, primary productivity satellite images) into g of jellyfish (wet weight) per 100 m^3^. The reported jellyfish density in the Pacific ranged from 1 g jellyfish m^−3^ (eastern North Pacific) to 100 g jellyfish m^−3^ (western Pacific) [75]. The densities reported by Lilley *et al*. [75] are 2–200 times greater than Lynam *et al*. [63] and could support the entire Pacific population of leatherbacks' yearly consumption in 2.1×10^10^−2.1×10^12^ m^3^, equivalent to 110–11,000 km^2^ (when considering an epipelagic depth of 200 m). However, Lilley *et al*. [75] do not report on species stating that their biomass estimates include epipelagic gelatinous zooplankton (i.e., scyphomedusae, hydromedusae, ctenophores, tunicates). Purcell *et al*. [76] reported *Aurelia sp.* aggregations in the North Pacific numbering in the hundreds to millions (known leatherback forage). How stable these jellyfish aggregations are, however, is unknown and it seems more likely that, in the ocean, seasonal and spatial fluctuations in jellyfish densities [64] will occur. As such, for the same amount of energy consumed, whilst in oceanic waters, leatherbacks probably expend more energy migrating between food patches than when inhabiting the coastal zones. With the reports of increases in gelatinous zooplankton [63], [64], [74] and reductions in leatherback population numbers [1], it is hard to conceive that leatherback recovery in the Pacific could be resource limited [6], [7]. To meet the resource requirements at the individual or population level, however, requires the dynamic meshing of the prey landscape in time and space with the needs of the individuals throughout their life cycle.

Model estimates suggest juveniles (2–7 years of age) account for the largest portion of the Pacific leatherback population's biomass (51%; 97,000 turtles) and food consumption (1.1×10^6^ t of jellyfish per year; 52%). According to the growth rate estimates and derived length-mass relationship of Jones *et al.* [29], a 7-year-old juvenile would be >100 cm SCL, ∼115 kg with an MR of 0.6 W kg^−1^ (derived from Fig. 3). By using this mass and MR in the thermoregulatory model of Bostrom & Jones [77] and Bostrom *et al.* [78], turtles of this size would be capable of maintaining a thermal gradient between body and ambient water temperature of 2–6°C. Animals of this size would therefore be confined to warmer, less-productive waters of the subtropical and southern temperate oceans. In these waters, juvenile and subadult leatherbacks, needing to consume 20 t of jellyfish a year (55 kg day^−1^), would be restricted to coastal areas or equatorial convergence zones [15], [16]. Unfortunately, coastal areas are associated with the highest registered mortality rates for marine turtles [4], [5], [79]. Even in oceanic waters, where mortality rates are lower, commercial fisheries tend to focus their efforts in the tropics [80], the same area leatherbacks probably congregate to find gelatinous prey [15], [16], [29], [81].

We assumed digestive efficiency (DE) of jellyfish to be 80%, as did Wallace *et al.* [6] and Hatase and Tsukamoto [50] who based their DE on a study of slider turtles [82] that were fed a diet high in protein [83]. To account for the nitrogenous loss in urine we modeled assimilation efficiency (AE) to be an additional 80% (total available energy 63%) based on a study of free-ranging lizards eating high protein diets [54], see Jones and Seminoff [84] for review of assimilation and digestion efficiency in sea turtles. Jellyfish and gelatinous zooplankton (scyphomedusae, hydromedusae, ctenophores, and tunicates) are also rich in mucopolysaccharides, long chains of sugars that can be hard to digest [33]. Therefore, direct studies of assimilation in leatherbacks for their various gelatinous prey types (Cnidarians, Ctenophores, and tunicates) are needed. Furthermore, as jellyfish species proportions, along with their environmental landscapes, are changing [63], [64] it will be important to determine if leatherbacks actually select scyphomedusae over hydromedusae or other gelatinous prey. Simple behavioral experiments such as those used by Constantino & Salmon [85] to determine the role of visual and chemical cues in hatchlings (i.e. circle tanks with tethered turtles attached to directional indicators) could be used to determine plasticity in leatherback prey choice as well as whether leatherbacks feed selectively on higher energy portions of jellyfish such as the oral arm or gonads [31]. Doyle *et al*. [31] determined the energy densities for 3 species of scyphomedusae and this type of study needs to be extended to include hydromedusae, ctenophores, and tunicates. Synthesizing these data on assimilation efficiency, prey selectivity, and energy densities of prey will provide a more complete picture of how changing jellyfish landscapes [13], [64] will affect leatherback ecology.

The calculated consumption to biomass ratio (*Q/B*, 96) for the Pacific leatherback population is 14 to 27 times greater than estimates for olive ridley (*Lepidochelys olivacea*), loggerhead (*Caretta caretta*), and green turtles (*Chelonia mydas*) of the eastern Tropical Pacific (3.5) [86] and for hawksbills (*Eretmochelys imbricata*) and greens off the Hawaiian Islands (3.5 and 6.8, respectively) [87]. The simplest explanation for the dichotomy in *Q/B* estimates of Dermochelyid and Cheloniid turtles is the energy density of their diet. Jellyfish have energy densities of 0.1–0.2 kJ g^−1^ WM [31], whereas the known diet of the Cheloniids (crustaceans, mollusks, seagrass) have energy densities of 2.0–6.0 kJ g^−1^ WM [11], [88]. If leatherbacks foraged on the same items as the Cheloniids their *Q/B* would be reduced to 3.2–9.6. Spotted and mesopelagic dolphins of the eastern tropical Pacific have increased MRs but lower *Q/B* ratios (16.5; [86]) than leatherbacks, while the dolphins require higher energy intake day^−1^ kg^−1^ their forage is nearly two orders of magnitude greater in energy density (7 kJ g^−1^ WM; [88]) explaining the lower *Q/B*.

Leatherbacks are large (upwards of 500 kg) gelatinous zooplanktivores with consumption to biomass ratios of 96 (up to 192 if feeding strictly on low energy density Cnidarians); they, therefore, have a large capacity to impact gelatinous zooplankton landscapes. Consequently, it is possible that leatherbacks have a much larger role to play in the ecosystem; were mature leatherbacks to be restored to abundance levels approximated to be common two decades ago (∼180,000), we estimate that the Pacific population would consume upwards of 61×10^6^ t of jellyfish per year. This intake would require foraging over 580,000 square kilometers at jellyfish densities reported by Lynam *et al*. [63]. Large pelagics such as leatherbacks and the sunfish (*Mola mola*) play a crucial role in reducing jellyfish numbers [89]. Warming climate patterns [64] and overfishing [60] may be leading to ecosystem changes where jellyfish are replacing fish as the dominant species [63], [90]. Restoration of leatherbacks to pre-1980 abundance could reduce the numbers of gelatinous zooplankton which can outcompete fish for resources and prey directly on fish eggs and larvae [62], [63]. Knowledge of ontogenetic resource requirements of leatherback turtles has applications in studies of population-level climate forcing (e.g., [91]). And understanding the leatherback's needs for gelatinous zooplankton, versus the availability of these resources, can help us better assess population trends and conservation status.
